# Synergistic nanoarchitecture of mesoporous carbon and carbon nanotubes for lithium–oxygen batteries

**DOI:** 10.1186/s40580-021-00268-5

**Published:** 2021-06-07

**Authors:** Yeongsu Kim, Jonghyeok Yun, Hyun-Seop Shin, Kyu-Nam Jung, Jong-Won Lee

**Affiliations:** 1 Department of Materials Science and Engineering, Chosun University, 309 Pilmun-daero, Dong- gu, Gwangju, 61452 Republic of Korea; 2 Department of Energy Science and Engineering, Daegu Gyeongbuk Institute of Science and Technology (DGIST), 333 Techno Jungang-daero, Hyeonpung-eup, Dalseong-gun, Daegu, 42988 Republic of Korea; 3 New and Renewable Energy Institute, Korea Institute of Energy Research, 152 Gajeong-ro, Yuseong-gu, Daejeon, 34129 Republic of Korea; 4 Energy Science and Engineering Research Center, Daegu Gyeongbuk Institute of Science and Technology (DGIST), 333 Techno Jungang-daero, Hyeonpung-eup, Dalseong-gun, Daegu, 42988 Republic of Korea

**Keywords:** Lithium–oxygen battery, Mesoporous carbon, Carbon nanotube, Electrochemistry

## Abstract

A rechargeable lithium–oxygen battery (LOB) operates via the electrochemical formation and decomposition of solid-state Li_2_O_2_ on the cathode. The rational design of the cathode nanoarchitectures is thus required to realize high-energy-density and long-cycling LOBs. Here, we propose a cathode nanoarchitecture for LOBs, which is composed of mesoporous carbon (MPC) integrated with carbon nanotubes (CNTs). The proposed design has the advantages of the two components. MPC provides sufficient active sites for the electrochemical reactions and free space for Li_2_O_2_ storage, while CNT forests serve as conductive pathways for electron and offer additional reaction sites. Results show that the synergistic architecture of MPC and CNTs leads to improvements in the capacity (~ 18,400 mAh g^− 1^), rate capability, and cyclability (~ 200 cycles) of the CNT-integrated MPC cathode in comparison with MPC.

**Supplementary Information:**

The online version contains supplementary material available at 10.1186/s40580-021-00268-5.

## Introduction

Recently, extensive efforts have been devoted for the development of high-energy-density batteries that came along with the explosive market expansion of electric vehicles (EVs) [1, 2]. Among the various battery types, lithium–oxygen batteries (LOBs) have been regarded as an alternative system for the state-of-the-art Li-ion batteries (LIBs) for long-range EV applications because of the expected higher energy densities of LOBs than those of LIBs [3–7]. Considering that LOBs can operate under ambient air containing CO_2_, there has recently been much attention on Li–O_2_/CO_2_ batteries for CO_2_ capture and utilization [8, 9]. In the LOB configuration with an aprotic electrolyte, it is known that the solid discharge products of Li_2_O_2_ (or Li_2_CO_3_) can be formed via the electrochemical reaction between Li^+^ and O_2_ (or CO_2_) supplied from ambient air, after which they can reversibly decompose upon subsequent charging as follows: 2Li^+^ + O_2_ + 2e^−^ = Li_2_O_2_ (or 4Li^+^ + O_2_ + 2CO_2_ + 4e^−^ = 2Li_2_CO_3_) [3, 8].

The cathode is a key component of LOBs that provides active sites to facilitate electrochemical reactions and accommodate solid discharge products (Li_2_O_2_ or Li_2_CO_3_) [10–15]. Thus, carbon has been considered to be viable cathode materials for LOBs that satisfy technical requirements such as large surface area, well-defined pore structure, high electrical conductivity, and low cost [3, 11]. Particularly, nanostructured porous carbon materials have attracted significant attention as promising cathodes for LOBs owing to their unique architectures, as they can offer sufficient pore volume for oxygen transport and Li_2_O_2_ storage, and have the high surface areas with many electrochemical active sites for oxygen reactions [3, 11, 16–19].

Guo et al. demonstrated the promising electrochemical performance of an LOB constructed with ordered hierarchical mesoporous/macroporous carbon [16]. They proposed that the hierarchical carbon nanoarchitectures not only facilitate Li^+^ diffusion but also offer void space for O_2_ transport and electrochemical reactions. Metal–organic frameworks (MOFs) with diverse pore structures have been demonstrated as potential cathode materials by Wu et al. stating that the open sites in the MOFs may allow for facile access of reactants, resulting in the high capacity and reversibility of LOBs [17]. Xie et al. reported an LOB cathode based on three-dimensional ordered mesoporous (3DOm) carbon [18]. It was suggested that Li_2_O_2_ was formed preferentially in the mesopores of the 3DOm carbon during discharge, thereby achieving a high capacity for LOBs. Recently, Lee and Park reported dual-phasic carbon nanoarchitectures, in which highly porous carbon nanoparticles derived from MOFs were interwoven with conductive carbon nanotubes (CNTs) as promising cathodes for LOBs [19].

Herein, we present a carbon-based LOB cathode, in which mesoporous carbon (MPC) and CNTs (MPC@CNT) are interconnected. The approach proposed in this study is to create a nanoarchitecture via the direct integration of CNTs on MPC nanoparticles. This nanoarchitecture allows us to incorporate the inherent characteristics of both MPC and CNTs. It was found that the nanostructured cathode exhibited improved electrochemical performance (capacity, rate capability, and cyclability) owing to the synergistic architecture of MPC and CNTs.

## Methods/experimental

### Material synthesis

The MPC material employed in this study was CNovel™ obtained from Toyo Tanso (Japan). CNTs were deposited onto MPC as follows. First, Co(NO_3_)_2_·6H_2_O (Sigma-Aldrich, 98%) was dissolved in deionized water. Subsequently, MPC powder was immersed in the solution, followed by drying at 90 ºC. The dried sample was placed in a covered crucible containing dicyandiamide (DCDA) (Sigma-Aldrich, 99%) in a tube furnace, which was then heated at 400 ºC for 3 h and at 800 ºC for 1 h in N_2_. Finally, the synthesized material was soaked in a 1 M H_2_SO_4_ solution for 24 h to remove excess Co species and MPC@CNT was obtained.

### Material characterizations

The morphologies and microstructures of the samples were characterized by scanning electron microscopy (SEM) (Hitach X–4900) and transmission electron microscopy (TEM) (Hitachi, HF-3300). X-ray diffraction (XRD) analysis was carried out using an X-ray diffractometer (2500 D/MAX, Rigaku), which utilizes a monochromatic Cu *K*_α_ radiation (*λ* = 1.5405 Å). The surface chemistry was examined by energy-dispersive X-ray spectroscopy (EDS) (Horiba) and X-ray photoelectron spectroscopy (XPS) (Thermo MultiLab 2000 spectrometer).

### Electrochemical experiments

To fabricate cathodes for LOBs, the suspension of MPC@CNT was prepared using deionized water and was coated directly onto a glass-fiber membrane (1.2 μm pore diameter) via vacuum-assisted filtration, as reported in our previous study [19]. Neither a polymeric binder nor a conductive agent was used. The prepared cathode had the area and mass loading of 0.785 cm^2^ and 1.0 mg cm^− 2^, respectively. The electrolyte was 1 M lithium bis(trifluoromethanesulfonyl)imide (LiTFSI) in tetraethylene glycol dimethyl ether (TEGDME). The electrochemical performance of the LOBs was evaluated by constructing 2032-type coin cells. After drying under vacuum at 120 °C for 24 h, the cell components were assembled in an Ar-filled glove box. The cell was composed of Li metal (anode), MPC@CNT (cathode)-coated glass-fiber membrane (separator) soaked with the electrolyte, and porous Ni foam (cathode current collector). High-purity O_2_ or O_2_/CO_2_ (1:2 in volume) gas was supplied to the cell. Discharge–charge measurements were conducted using a battery test system (WonATech, WBCS3000L32) at room temperature. The cell was discharged to 500 mAh g^− 1^, and then, it was charged at a constant current density to either 4.3 or 4.5 V vs. Li/Li^+^, depending on the atmosphere, followed by constant voltage charging with a 50 mA g^− 1^ cut-off current or a 500 mAh g^− 1^ cut-off capacity, whichever occurred first. The potentiostatic intermittent titration technique (PITT) was employed to obtain quasi-equilibrium potentials of LOBs during charging as follows [20, 21]: the cell was discharged to 500 mAh g^− 1^ at 50 mA g^− 1^ and was charged by applying a voltage step of 12 mV until a steady state current was obtained. The PITT step was repeated until the capacity reached 500 mAh g^− 1^. AC impedance spectra were measured at frequency values of 10^− 2^–10^6^ Hz (5 mV amplitude) using a Bio-Logic SP-200.

## Results and discussion

**Fig. 1:**
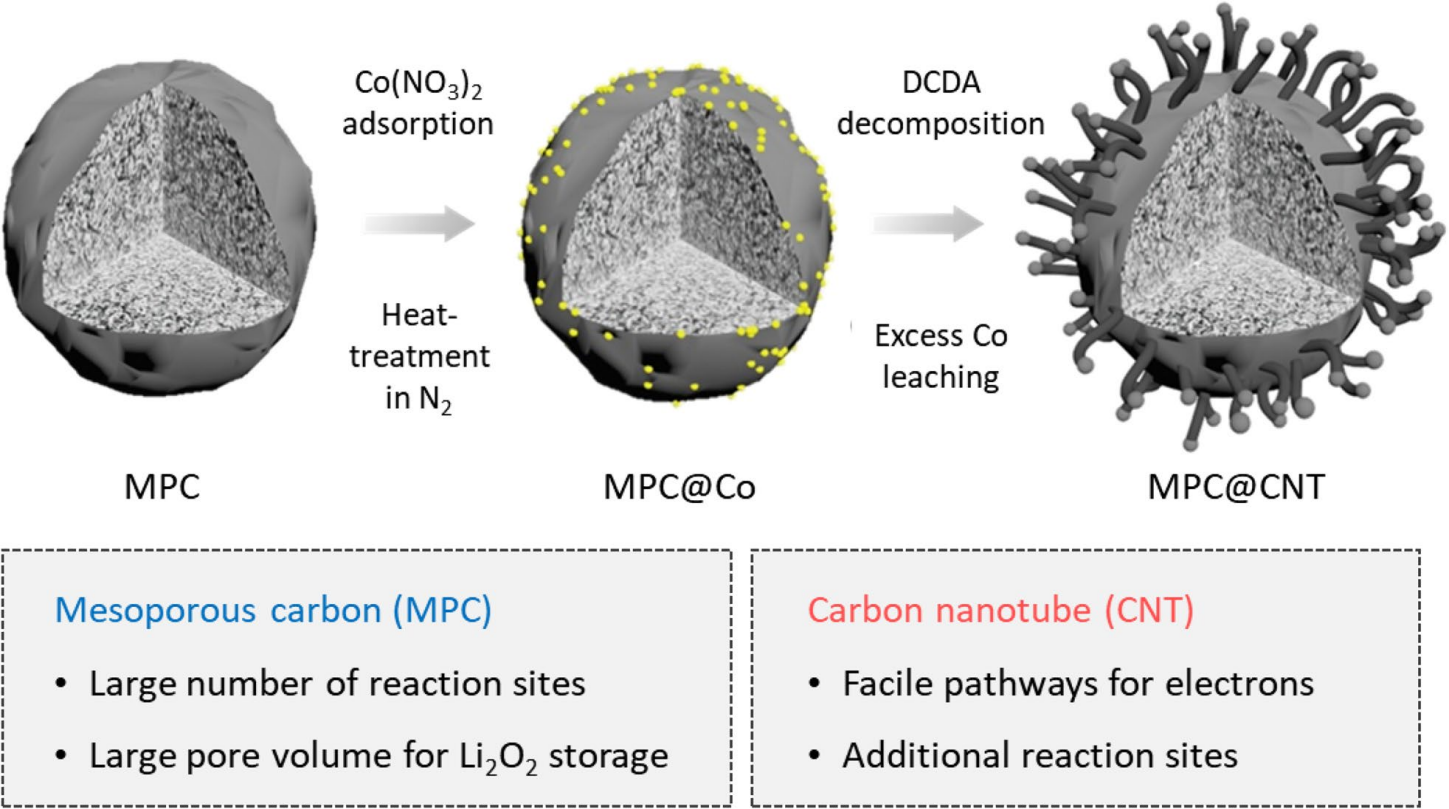
Schematic diagram illustrating the synthesis process of MPC@CNT

The fabrication process for MPC@CNT is schematically illustrated in Fig. 1. With the help of Co seeds, one-dimensional (1D) CNTs were grown directly on the surface of MPC via thermal decomposition of DCDA in N_2_. The DCDA has been known as a useful precursor for fabricating CNT-based nanoarchitectures, e.g., Co-embedded N‐rich CNTs [22] and N‐doped graphene/graphene‐tube nanocomposites [23]. After that, the MPC@CNT material was treated with H_2_SO_4_ to etch out excess Co species. As a cathode for LOBs, this nanostrucutre provides the adavtantages of both MPC and CNT. MPC has a high surface area (~ 1,685 m^2^ g^− 1^) for the electrochemical reactions and a large free space available for Li_2_O_2_ accommodation. 1D CNTs offer conductive pathways for electron and additional electrochemically active sites on the MPC surface.

**Fig. 2:**
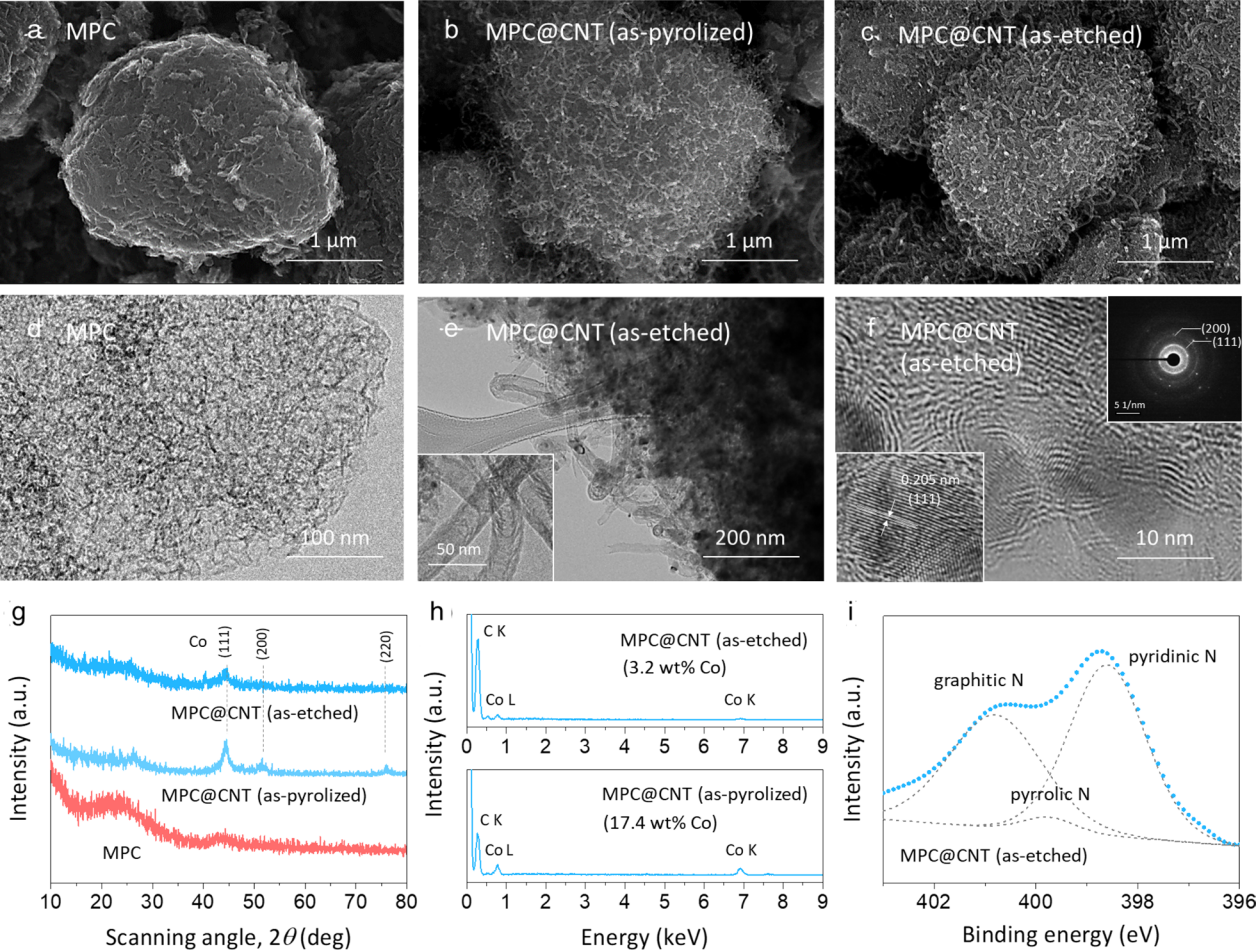
SEM images of **a** MPC, **b** as-pyrolized MPC@CNT, and **c** as-etched MPC@CNT. TEM images of **d** MPC and **e, f** as-etched MPC@CNT. The SAED pattern is shown in the inset of **f**. **g** XRD and **h** EDS of the synthesized samples. **i** XPS N 1* s* spectrum for MPC@CNT

The microstructural details of the synthesized MPC@CNT were investigated using various characterization tools, as shown in Fig. 2. Figure 2a–c present the SEM micrographs of pristine MPC, as-pyrolized MPC@CNT, and as-etched MPC@CNT, respectively. The key experimental finding was that MPC particles with diameters of 3–5 μm retained its original morphology without significant microstructural changes after the formation of CNTs and chemical etching. 1D CNT forests were grown conformally over the entire MPC particles. Hence, they may provide additional active sites for oxygen reactions on the MPC particles and enable facile conduction of electron during LOB operations. Furthermore, the CNTs were in direct contact with the MPC, thereby reducing the contact resistance between them. TEM analysis (Fig. 2d–f) indicated that MPC possessed a well-defined mesoporous structure with interconnected pores (approximaterly ten to tens of micrometers), whereas in MPC@CNT, the MPC surface was decorated with CNT forests (thickness ~ 30 nm).

XRD and TEM analyses revealed the presence of residual Co species in the MPC@CNT. As shown in Fig. 2g, the diffraction peaks of the typical Co phase were detected at 2*θ* = 44.4, 51.5, and 76.1°, which can be indexed to (111), (200), and (220) planes of crystalline Co phase, respectively [24]. The lattice fringe spacing, *d* = 0.205 nm, was observed in the TEM image (Fig. 2f), which was assigned to the (111) crystalline plane of the Co phase. Furthermore, the selected area electron diffraction (SAED) pattern (inset in Fig. 2f) shows the characteristic diffraction rings for the (111) and (200) planes of the Co phase, which is consistent with the XRD results. EDS analysis (Fig. 2h) confirmed that the intensities of the diffraction peaks for the Co phase greatly reduced after the chemical etching process. Metallic Co species might be dissolved into the electrolyte and/or be vulnerable to chemical attack by oxygen radicals during LOB operations. However, the negative impact of Co would be minimal because any unstable Co species were etched by a strong acidic solution and most of Co species after etching were encapsulated by thin graphene layers (Fig. 2f).

The surface chemistry of MPC@CNT (as-etched) was further examined via XPS, and the results are shown in Fig. 2i. The N 1* s* spectrum features three components at binding energies of 398.6, 399.8 and 400.9 eV that correspond to pyridinic N, pyrrolic N, and graphitic N, respectively [19]. The electronic conductivity of carbon can be improved greatly by N doping, which alters the band structure of carbon [25, 26]. Moreover, previous studies on the catalysis of the fuel cells and metal–O_2_ batteries suggest that the pyridinic and pyrrolic N species are strongly responsible for facilitating oxygen reactions on carbon [27–30]. Thus, MPC@CNT is expected to have more desirable surface conditions that can improve electron conduction and oxygen reactions.

**Fig. 3:**
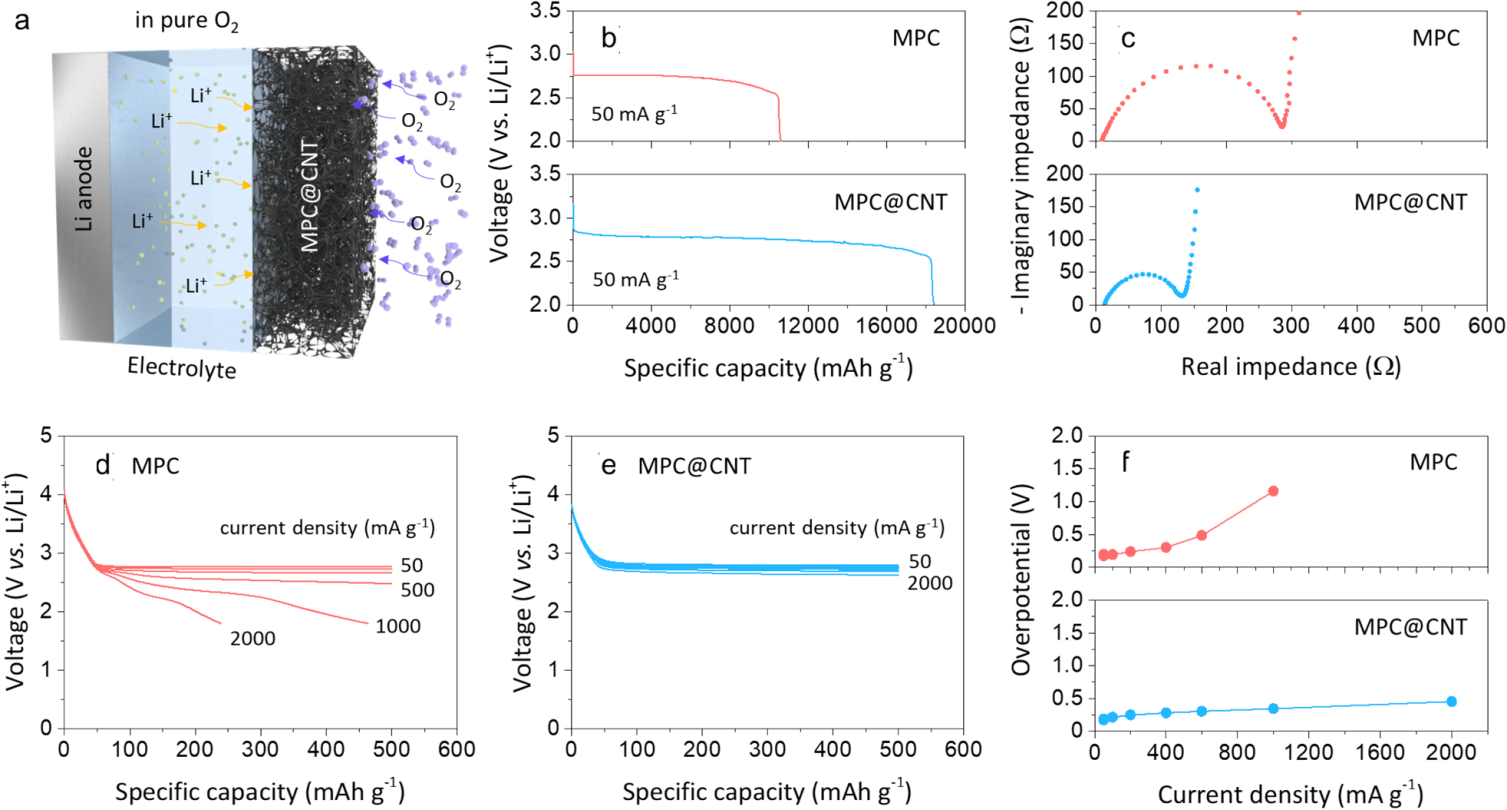
**a** Schematic diagram for the LOB operating in pure O_2_. **b** Galvanostatic discharge profiles and **c** AC-impedance spectra for the LOBs with fresh MPC and MPC@CNT. Discharge profiles of the LOBs with **d** MPC and **e** MPC@CNT measured at various current densities and **f** the plots of overpotential vs. current density obtained from the rate performance tests

To compare the electrochemical performances of MPC and MPC@CNT, LOBs were constructed and tested in pure O_2_ atmosphere (Fig. 3a). Figure 3b presents typical discharge curves of the LOBs assembled with MPC and MPC@CNT. The curves were acquired at 50 mA g^− 1^. Both cathodes exhibited wide voltage plateaus at ~ 2.7 V vs. Li/Li^+^, subsequently followed by abrupt decline to 2.0 V vs. Li/Li^+^. Compared with the MPC-only electrode (~ 10,560 mAh g^− 1^), the MPC@CNT electrode delivered a capacity as high as 18,400 mAh g^− 1^, demonstrating the beneficial role of CNTs in enhancing the achievable capacity. The SEM (Additional file 1: Figure S1) and XPS (Additional file 1: Figure S2) characterizations confirmed the formation of Li_2_O_2_ on the cathode upon discharge. The AC-impedance spectra of LOBs with MPC and MPC@CNT are presented in Fig. 3c. Both of AC impedance spectra exhibit a slightly depressed semicircle in the high frequency range and a straight line in the low frequency range, which can be assigned to the interfacial redox reactions and the finite-length gas diffusion, respectively [5, 12, 31]. It should be noted that the interfacial resistance of MPC@CNT was estimated to be 285 Ω, which is much lower than that of MPC (131 Ω). This implies the improved interfacial kinetics of Li_2_O_2_ formation–decomposition on MPC@CNT in comparison with the MPC-only electrode.

To examine the rate performance of MPC@CNT, the cathodic overpotential was evaluated at various current densities during discharge. Here, the overpotential was determined as the difference between the theoretical redox potential (2.96 V vs. Li/Li^+^) and the terminal voltage measured with a fixed capacity of 500 mAh g^− 1^ at a given applied current density [19, 32]. Figure 3d and e show the galvanostatic discharge profiles of the LOBs with MPC and MPC@CNT, respectively, obtained by applying various current densities in the range of 50 to 2000 mA g^− 1^. Notably, the LOB with MPC@CNT can easily reach 500 mAh g^− 1^ without significant polarization over the entire current range, while the LOB with MPC showed a highly polarized curve shape at current densities above 1000 mA g^− 1^, and exhibited sudden decay (delivered capacity of 240 mAh g^− 1^) at 2000 mA g^− 1^. The benefits of the integration between MPC and CNT for improving the rate capability can be confirmed because the LOB with MPC@CNT has considerably lower overpotentials than the LOB with MPC, as shown in Fig. 3f. The reduced overpotential and enhanced rate capability of the MPC@CNT electrode are mainly attributed to the higher conducting properties of CNTs decorated on MPC particles.

**Fig. 4:**
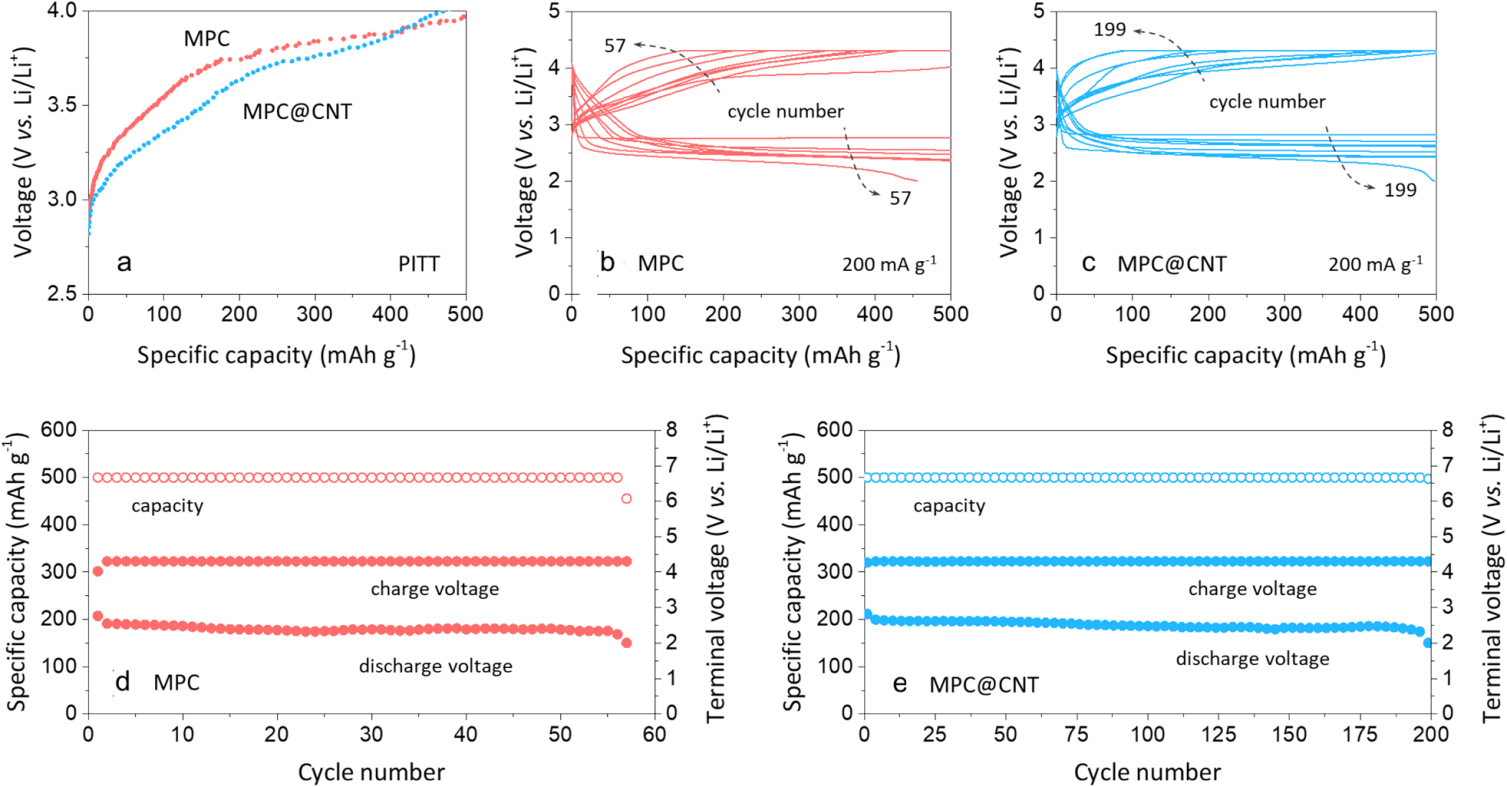
**a** Quasi-equilibriium charge potential profiles of the LOBs with MPC and MPC@CNT obtained via PITT experiments. Discharge–charge curves of the LOBs with **b** MPC and **c** MPC@CNT during cycling at 200 mA g^− 1^. Plots of capacity and terminal (discharge/charge) voltages against the cycle number for the LOBs with **d** MPC and **e** MPC@CNT

To further examine the charging behavior of LOBs assembled with MPC@CNT, the PITT experiment was carried out under pure O_2_. As demonstrated in previous studies [12, 21], PITT analysis provided useful information on the charging characteristics of LOBs under quasi-equilibrium conditions by imposing a series of small anodic potential steps to drive the decomposition of reaction products. For the PITT, the LOB was first discharged to 500 mAh g^− 1^ at 50 mA g^− 1^, followed by PITT charging with a 12 mV step. Figure 4a presents the charge profiles of the LOBs with MPC and MPC@CNT under pure O_2_ conditions. It was observed that the quasi-equilibrium charge potentials of the LOB with MPC@CNT are lower than those of the LOB with MPC over the entire capacity range. This means that the decorated CNTs play a beneficial role in facilitating the electrochemical decomposition of Li_2_O_2_, which may lead to the improved cyclability as discussed below.

The cycling performances of the LOBs assembled with the MPC-only and MPC@CNT cathodes under pure O_2_ were compared. The LOBs were cycled with 500 mAh g^− 1^ at 200 mA g^− 1^. Figure 4b and c display the discharge–charge curves of the LOBs with MPC and MPC@CNT at selected cycles, respectively. It is observed that the voltage profiles of the LOB with the MPC-only cathode exhibited significant and abrupt changes in comparison with the LOB with MPC@CNT. As discussed from the PITT results, the LOB with MPC@CNT showed considerably improved cycling stability (~ 200 cycles) as compared to the LOB constructed with the MPC*-*only electrode, which exhibited early cycling instability after 57 cycles (Fig. 4d and e). The cell failure is believed to be due to the parasitic reactions on the cathode (carbon degradation and electrolyte decomposition), resulting in a continuous build-up of resistive components over the course of cycling [3].

**Fig. 5:**
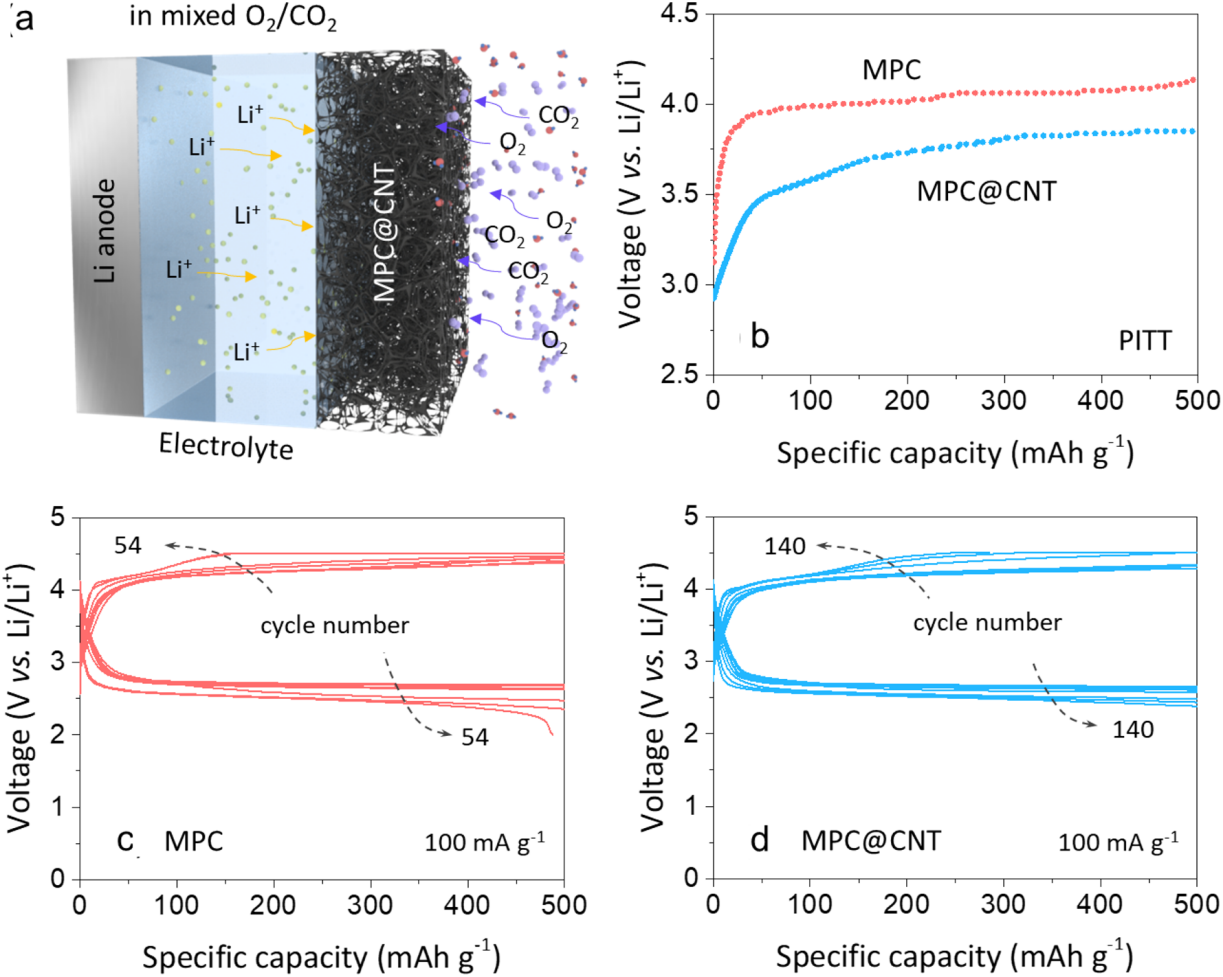
**a** Schematic diagram for the LOB operating in a mixed O_2_/CO_2_ atmosphere. **b** Quasi-equilibriium charge potential profiles of the LOBs with MPC and MPC@CNT obtained via PITT experiments. Discharge–charge curves of the LOBs with **c** MPC and **d** MPC@CNT during cycling at 100 mA g^− 1^

Given that LOB systems may be exposed to an ambient atmosphere containing CO_2_, the cell tests were conducted in a mixed gas of O_2_ and CO_2_ (1:2 in volume) to determine the feasibility in the presence of CO_2_ (Fig. 5a). Both of the LOBs showed higher charging potentials in the mixed O_2_/CO_2_ atmosphere than in pure O_2_, which agrees with the fact that the formation of Li_2_CO_3_ causes a significant increase in the interfacial resistance to charge transfer, resulting in the higher charging potentials of LOBs [3]. Similarly, for the O_2_/CO_2_ condition, the quasi-equilibrium charge potentials of the LOB with MPC@CNT were observed to be lower than those of the LOB with MPC, which exhibited a wide potential plateau over 4.0 V vs. Li/Li^+^ below where only 200 mAh g^− 1^ was charged (Fig. 5b). These PITT results strongly indicate the effectiveness of CNT integration for promoting the decomposition of Li_2_CO_3_ as well as Li_2_O_2_, thus improving the reversibility of the electrochemical reaction. As shown in Fig. 5c, d, in fact, the LOB with MPC@CNT exhibited an improvement in the cycling stability (> 140 cycles) at 100 mA g^− 1^, when compared to the LOB with MPC (~ 54 cycles), under the O_2_/CO_2_ condition.

## Conclusions

In this study, a carbon nanoarchitecture for LOB cathodes in which mesoporous MPC particles were integrated with conductive CNT forests were designed and synthesized. The MPC@CNT material was synthesized by the direct formation of CNTs on MPC via a facile vapor deposition process combined with chemical etching. The LOB with MPC@CNT exhibited a capacity of ~ 18,400 mAh g^− 1^ and high rate capability in pure O_2_. In particular, the MPC@CNT electrode showed improved interfacial kinetics in comparison with the MPC-only electrode, thus enhancing the cyclability of the LOBs under both O_2_ and O_2_/CO_2_ conditions. The improved electrochemical performance of MPC@CNT results from the synergistic role of MPC and CNT: (i) highly mesoporous MPC provides a large number of active sites for the electrochemical O_2_ and CO_2_ reactions and sufficient free space for discharge products; and (ii) highly conductive 1D CNTs integrated on MPC particles secure facile pathways for electrons and offer additional active sites.

## Supplementary Information

**Additional file: Figure S1.** SEM micrograph of the MPC@CNT cathode after discharge in O_2_. **Figure S2.** Li 1s XPS spectrum of the discharged MPC@CNT cathode in O_2_.

## Data Availability

The datasets used and/or analyzed during the current study are available from the corresponding author on reasonable request.
