# Supplementary material for: Synergistic nanoarchitecture of mesoporous carbon and carbon nanotubes for lithium–oxygen batteries
Source: Nano Converg. 2021 Jun 7;8:17. doi: 10.1186/s40580-021-00268-5 (PMC8184898; doi:10.1186/s40580-021-00268-5)
Supplement: Supplementary file 1 — Additional file: Figure S1. SEM micrograph of the MPC@CNT cathode after discharge in O2. Figure S2. Li 1s XPS spectrum of the discharged MPC@CNT cathode in O2. [file 40580_2021_268_MOESM1_ESM.docx]

**Additional Information**

**Synergistic nanoarchitecture of mesoporous carbon and carbon nanotubes for lithium–oxygen batteries**

Yeongsu Kim^1^, Jonghyeok Yun^2^, Hyun-Seop Shin^3^, Kyu-Nam Jung^3,*^, Jong-Won Lee^2,4,*^

^1^ Department of Materials Science and Engineering, Chosun University, 309 Pilmun-daero, Dong-gu, Gwangju 61452, Republic of Korea

^2^ Department of Energy Science and Engineering, Daegu Gyeongbuk Institute of Science and Technology (DGIST), 333 Techno Jungang-daero, Hyeonpung-eup, Dalseong-gun, Daegu 42988, Republic of Korea

^3^ New and Renewable Energy Institute, Korea Institute of Energy Research, 152 Gajeong-ro, Yuseong-gu, Daejeon 34129, Republic of Korea

^4^ Energy Science and Engineering Research Center, Daegu Gyeongbuk Institute of Science and Technology (DGIST), 333 Techno Jungang-daero, Hyeonpung-eup, Dalseong-gun, Daegu 42988, Republic of Korea

* Corresponding author: Kyu-Nam Jung

Tel.: +82-42-860-3617

E-mail address: mitamire@kier.re.kr

* Corresponding author: Jong-Won Lee

Tel.: +82-53-785-6430

E-mail address: jongwon@dgist.ac.kr


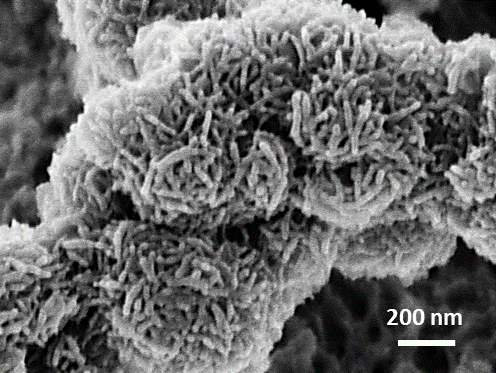


**Fig. S1** SEM micrograph of the MPC@CNT cathode after discharge in O_2_.


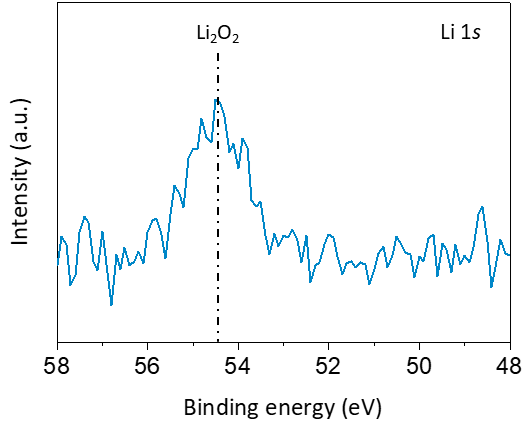


**Fig. S2** Li 1*s* XPS spectrum of the discharged MPC@CNT cathode in O_2_.
